# Association and pathways between shift work and cardiovascular disease: a prospective cohort study of 238 661 participants from UK Biobank

**DOI:** 10.1093/ije/dyab144

**Published:** 2021-08-20

**Authors:** Frederick K Ho, Carlos Celis-Morales, Stuart R Gray, Evangelia Demou, Daniel Mackay, Paul Welsh, S Vittal Katikireddi, Naveed Sattar, Jill P Pell

**Affiliations:** Institute of Health and Wellbeing, University of Glasgow, Glasgow, UK; Institute of Health and Wellbeing, University of Glasgow, Glasgow, UK; Institute of Cardiovascular and Medical Sciences, University of Glasgow, Glasgow, UK; Institute of Cardiovascular and Medical Sciences, University of Glasgow, Glasgow, UK; Institute of Health and Wellbeing, University of Glasgow, Glasgow, UK; Institute of Health and Wellbeing, University of Glasgow, Glasgow, UK; Institute of Cardiovascular and Medical Sciences, University of Glasgow, Glasgow, UK; Institute of Health and Wellbeing, University of Glasgow, Glasgow, UK; Institute of Cardiovascular and Medical Sciences, University of Glasgow, Glasgow, UK; Institute of Health and Wellbeing, University of Glasgow, Glasgow, UK

**Keywords:** Shift work, mediation, cardiovascular disease

## Abstract

**Background:**

This study aimed to study the association between shift work and incident and fatal cardiovascular disease (CVD), and to explore modifying and mediating factors.

**Methods:**

This is a population-based, prospective cohort study with a median follow-up of 11 years; 238 661 UK Biobank participants who were in paid employment or self-employed at baseline assessment were included.

**Results:**

Shift workers had higher risk of incident [hazard ratio (HR) 1.11, 95% confidence interval (CI) 1.06–1.19] and fatal (HR 1.25, 95% CI 1.08–1.44) CVD compared with non-shift workers, after adjusting for socio-economic and work-related factors. The risk was higher with longer duration of shift work, in women and in jobs with little heavy manual labour. Current smoking, short sleep duration, poor sleep quality, adiposity, higher glycated haemoglobin and higher cystatin C were identified as the main potentially modifiable mediators. Mediators collectively explained 52.3% of the associations between shift work and incident CVDs.

**Conclusions:**

Shift workers have higher risk of incident and fatal CVD, partly mediated through modifiable risk factors such as smoking, sleep duration and quality, adiposity and metabolic status. Workplace interventions targeting these mediators have the potential to alleviate shift workers’ CVD risk.

Key Messages

- Shift workers were found to have a higher risk of cardiovascular disease, but the existing studies had multiple sources of biases.
- This study addresses some of the existing biases and identified an elevated cardiovascular risk among shift workers, especially among people with longer duration of shift work, women and people in jobs with minimal heavy manual labour.
- Using a counterfactual framework, this study also identified current smoking, short sleep duration, poor sleep quality, adiposity, blood glucose and renal impairment were identified as the main potentially modifiable mediators.

## Introduction

Mortality rates vary greatly by occupation and shift work could be one of the contributing factors.^1^ Shift work often involves alternating work schedules and/or working in the evening, overnight or at weekends. It has become increasingly common in the service, transport and healthcare industries, and is undertaken by >25% of workers in England.^2^ The increasing prevalence of shift work is, in part, due to globalization and a 24-hour service culture.^3^ Shift work has been associated with a higher risk of various physical and mental health conditions,^4^^,^^5^ which has prompted discussions on whether shift work should be considered an occupational hazard for compensation purposes.^6^

A meta-analysis of 173 010 participants from 21 studies reported that shift workers were at 26% and 17% higher risk of ischaemic heart disease (IHD) and cardiovascular disease (CVD) mortality, respectively.^7^ Another large prospective cohort study of 189 158 women also illustrated an elevated work of IHD among those working in rotating night shifts.^8^ However, the mechanisms by which shift work may cause CVD are still elusive. Reviews have suggested behavioural, psychosocial and physiological pathways.^9^^,^^10^ Behavioural factors included sleep duration and quality, smoking, nutrition, body weight and physical activity; psychosocial factors included work stress and inability to attend social activities; and physiological factors included blood pressure, inflammation, and lipid and glucose metabolism. However, these hypotheses were largely based on limited direct evidence and their relative importance has not been well studied.

In addition, the meta-analysis,^7^ and its accompanying editorial,^11^ highlighted several important limitations of previous studies. First, some of the previous studies were subject to selection bias. Second, many previous studies treated shift work as a binary variable, ignoring the potential moderating effects of the type, frequency and period of shift work. Last, but not least, confounders and mediators were often not well defined or appropriately handled. On the one hand, the omission of socio-economic and work-related confounders (e.g. heavy manual work) may have resulted in residual confounding; on the other hand, adjusting for lifestyle factors such as obesity and smoking could have resulted in over-adjustment bias since they are likely to operate as mediators.^12^ As a result, only two of the studies included in the meta-analysis were categorized as having low risk of bias.

Therefore, this study aimed to investigate the associations between shift work and incident and fatal CVD with robust adjustment for confounders, supported by mediation and moderation analysis.

## Methods

### Study design and participants

UK Biobank is a prospective cohort study. Between 2007 and 2010, UK Biobank recruited 502 506 participants from the general population. Participants attended 1 of 22 assessment centres across England, Scotland and Wales where they completed a self-administered, touch-screen questionnaire and face-to-face interview, and trained staff took a series of measurements including: height, weight and blood pressure. This study included only the 287 141 participants who were in paid employment or self-employed. We also excluded 719 participants who did not answer the question on shift work and 47 753 participants who reported chronic conditions (including CVD, cancer, mental illnesses, etc.) at baseline to reduce reverse causation. The final study population comprised 238 661 participants (Supplementary Figure S1, available as Supplementary data at *IJE* online). All measurements in this study, except for CVDs, were measured at baseline assessment. CVDs were ascertained through record linkage and could occur from baseline assessment to the date of censoring.

### Employment

Participants were asked their employment status, length of current employment and number of hours worked per week. They were also asked to indicate whether their current job involves walking or standing, heavy manual or physical work, shift work and night-shift work on four-point Likert scales: never/rarely, sometimes, usually or always. In this study, the primary exposure variable of interest was any shift work, defined as participants who reported their job requiring them to sometimes, usually or always work shifts. No shift work was defined as jobs that rarely/never require shift work. Shift work was further categorized based on shift-work frequency (always vs sometimes/usually) and whether shifts were night shifts (shifts sometimes/usually/always at night) or evening/weekend shifts (never/rarely at night). The UK government defined night work as at least 3 hours of work during the period of 11 pm to 6 am,^13^ so participants who worked evening/weekend shifts may have worked until 11 pm with potentially ≤3 hours overnight. For participants who reported working shifts, the duration of the shift work was assumed to be the duration of their current employment.

### Outcomes

Outcomes were ascertained through individual-level record linkage of the UK Biobank cohort to routine administrative databases. Date and cause of death were obtained from death certificates held by the National Health Service Information Centre (England and Wales) and the National Health Service Central Register Scotland (Scotland). Dates and causes of hospital admissions were obtained through record linkage to Health Episode Statistics (England and Wales) and Scottish Morbidity Records (Scotland). Detailed information about the linkage procedures can be found at http://content.digital.nhs.uk/services. At the time of analysis, mortality data were up to 30 June 2020 and hospital-admission data were available up to 31 May 2020 for participants in England and 31 March 2017 for those in Scotland and Wales. We defined CVD as IHD [ICD-10 (International Classification of Diseases, 10th revision) codes I10–25], heart failure (I11.0, I42.0, I42.6–42.7, I42.9, I50) and stroke (I60–64).

### Potential mediators

Sleep duration (hours), television viewing (hours), smoking status and alcohol intake (units) were self-reported. Blood pressure (mmHg) was measured using automatic devices. Physical activity [total metabolic equivalent of task (MET) minutes] was self-reported using the validated International Physical Activity Questionnaire.^14^ Grip strength (kg), a marker of muscle weakness, was measured to the nearest 0.1 kg using a Jamar J00105 hydraulic hand dynamometer and the mean value from both hands used in the analyses. Height was measured to the nearest centimetre, using a Seca 202 stadiometer, and body weight to the nearest 0.1 kg, using a Tanita BC-418 body-composition analyser. Body mass index (BMI) was calculated as weight/height^2^ and the World Health Organization’s criteria were used to classify BMI into: underweight (<18.5 kg/m^2^), normal weight (18.5–24.9 kg/m^2^), overweight (25–29.9 kg/m^2^) and obese (≥30.0 kg/m^2^). BMI was categorized largely because of its nonlinear association with health outcomes. Central obesity was defined as waist–hip ratio >0.85 for women and >0.90 for men. Biomarkers were measured at a dedicated central laboratory between 2014 and 2017. Our analyses included low-density lipoprotein (LDL) cholesterol (mmol/L), lipoprotein(a) (nmol/L), glycated haemoglobin (mmol/mol), cystatin C (mg/L) and gamma-glutamyltransferase (GGT) (U/L) as potential mediators. LDL cholesterol and lipoprotein(a) were found to be causally related to CVD^15^ and to lifestyle factors such as smoking^16^ and obesity; glycated haemoglobin (HbA1c) is a marker of diabetes and related to obesity; cystatin C is a marker of kidney function and related to diet, smoking and body weight; and gamma-glutamyltransferase is a marker of liver function and related to alcohol, drinking and fatty liver disease. It should be noted that cystatin C and GGT were unlikely to be causal of CVD.^17–19^ However, they were used as proxy measures of chronic kidney and liver diseases in this study. The measurements of biomarkers were externally validated with stringent quality control.^20^

### Socio-demographic confounders

Ethnicity and highest level of education were self-reported. The Townsend area deprivation index was obtained from the postcode of residence and is derived using aggregated data on unemployment, car and home ownership, and household overcrowding.^21^

### Statistical analyses

Cox proportional-hazard models were used to analyse the association between shift work and CVD, with the results reported as hazard ratios (HRs) and 95% confidence intervals (CIs). The models were adjusted for age at baseline assessment, sex and ethnicity in Model 0, education level and deprivation index additionally in Model 1 and hours of work per week, duration of current job, walking/standing at work and heavy manual/physical work additionally in Model 2. These factors were chosen because they were plausible confounders. The associations with frequency and type of shift work were also estimated, with non-shift workers as the reference group. The relationship between years of shift work and CVD was analysed using a penalized cubic spline in a Cox model, adjusting for the same covariate, with no shift work (duration = 0 year) as the reference category. Penalized spline is a variation of basis spline, which is robust against the number of knots and knot placements.^22^

Subgroup analyses were conducted by socio-demographic factors: sex, age group (≤ and >50 years), education level (with and without university degree), area-based deprivation index (≥ and <median), hours of work per week (≤37 and >37), duration of current job (≤10 and >10 years), heavy manual work (never/rarely and sometimes/often) and chronotype (definitely morning/more morning than evening/more evening than morning and definitely evening). We selected these factors because these may be related to the type of shift work undertaken or may modify the association.

We studied eight groups of potential mediators of the association between shift work and CVD: physical activity [total MET-minutes per week, and hours of television viewing (continuous variables)], diet [portions of fruit/vegetables and red-meat intake per week (continuous variables), oily-fish intake (yes/no) and processed-meat intake (>2 and ≤2 times a week), smoking status (current and non-smoker), units of alcohol consumed per week continuous variables)], sleep duration (<6 and ≥6 hours per day) and number of sleep disturbances (difficulty getting up, insomnia, snoring, daytime sleepiness) (continuous variable), social factors [frequency of social visits (continuous variable)], adiposity [obesity and central obesity (binary variables)], physical markers [systolic blood pressure and handgrip strength (continuous variables)] and serum biomarkers [LDL cholesterol, lipoprotein(a), and glycated haemoglobin, cystatin C and gamma-glutamyltransferase (continuous variables)]. All potential mediators were selected a priori based on their plausible roles in the pathways between shift work and CVD as shown in the directed acyclic graph (Supplementary Figure S2, available as Supplementary data at *IJE* online). We assumed age, sex, education, area-based deprivation, hours of work per week, duration of current job, walking/standing at work and heavy manual/physical work to be the common set of confounders for exposure–outcome and mediator–outcome relationships. The groups of mediators were included in the Cox models to examine whether, and to what extent, the associations between shift work and CVD were attenuated as an exploration of mediation. Formal mediation analysis based on counterfactual framework was then conducted.^23^ Counterfactual framework formally defines direct (non-mediated) and indirect (mediated) effects, and are more robust against various limitations of traditional adjustment-based mediation analysis, such as mediator–outcome confounding affected by exposure.^24^ To reduce multicollinearity and unnecessary adjustment, the potential mediators were selected using a stepwise approach. First, CVD events were regressed by shift work and all potential mediators and confounders in a Weibull-regression model with robust standard errors. Weibull regression was chosen because of its superior statistical properties in mediation analysis.^25^ Potential mediators were then selected based on effect sizes and C statistics. The selected potential mediators were then regressed by shift work and other covariates (mediator model) in either logistic (for binary mediators) or multiple linear (for other mediators) models adjusting for other mediators and confounders. The outcome and mediator models were then combined to compute the natural indirect effect (NIE) and total effect (TE) for each participant, which was then averaged. Quasi-Bayesian estimation with 1000 iterations was used for estimating the 95% CI and *p*-values of the NIE and TE. Mediation proportion was calculated as NIE/TE.

Because biomarkers can lie on the pathways between lifestyle and CVD, the mediation analyses were replicated without them. This helped to identify lifestyle factors that might be masked by the adjustment for biomarkers. Proportional-hazard assumptions were verified by statistical tests based on Schoenfeld residuals. Age and sex violated the proportional-hazard assumption and were regarded as strata in subsequent analysis. The distributional assumption of Weibull regression was examined using the Kaplan–Meier estimate of the residuals, which revealed a close fit. Missing data were handled using complete case analysis. All analyses were conducted using R version 4.0.3 with packages *survival* and *mediation*.

## Results

Of the 238 661 participants analysed, 22 664 (9.5%) worked shifts sometimes or usually and 18 400 (7.7%) always worked shifts. Among shift workers, around half (*n* = 19 768) worked evening/weekend shifts and half (*n* = 21 234) worked night shifts. In general, shift workers were younger; more likely to be male, of non-White ethnicity and more deprived; worked longer hours; and did more walking and manual/physical labour at work (Table 1). They were more likely to smoke, were more physically active, consumed more red and processed meat and less oily fish, slept less, had more sleep disturbances and had higher BMI and waist circumferences.

**Table 1:** Characteristics of the cohort

	Overall	No shift work	Evening/weekend shift	Night shift
Total participants [*n*]	238 661	197 597	19 768	21 234
Mean (SD) age (years)	52.43 (7.04)	52.62 (7.05)	52.19 (7.01)	50.89 (6.73)
Male [*n* (%)]	116 507 (48.82)	93 340 (47.24)	9649 (48.81)	13 482 (63.49)
Ethnicity [*n* (%)]				
White	223 081 (93.74)	186 972 (94.89)	17 777 (90.21)	18 302 (86.51)
Mixed	1702 (0.72)	1326 (0.67)	179 (0.91)	197 (0.93)
South Asian	5158 (2.17)	3578 (1.82)	746 (3.79)	814 (3.85)
Black	4764 (2.00)	3010 (1.53)	575 (2.92)	1172 (5.54)
Chinese	955 (0.40)	741 (0.38)	98 (0.50)	115 (0.54)
Others	2313 (0.97)	1423 (0.72)	331 (1.68)	555 (2.62)
College or university degree [*n* (%)]	90 370 (38.20)	81 179 (41.44)	4901 (25.02)	4281 (20.32)
Mean (SD) deprivation index	−1.36 (3.00)	−1.53 (2.91)	−0.66 (3.23)	−0.50 (3.32)
Mean (SD) years working in current job	13.61 (10.52)	13.56 (10.52)	12.96 (10.20)	14.63 (10.76)
Mean (SD) number of work hours a week	35.63 (12.63)	35.07 (12.51)	36.04 (12.27)	40.51 (13.00)
Walking at work [*n* (%)]				
Never/rarely	84 466 (35.42)	78 564 (39.79)	3119 (15.79)	2779 (13.11)
Sometimes	73 068 (30.64)	61 866 (31.33)	5620 (28.46)	5563 (26.24)
Usually	35 141 (14.74)	26 324 (13.33)	3958 (20.04)	4845 (22.85)
Always	45 786 (19.20)	30 703 (15.55)	7051 (35.70)	8012 (37.79)
Heavy manual/physical labour at work [*n* (%)]				
Never/rarely	155 352 (65.14)	141 538 (71.67)	7773 (39.36)	6029 (28.43)
Sometimes	50 986 (21.38)	35 600 (18.03)	6985 (35.37)	8378 (39.51)
Usually	16 293 (6.83)	10 354 (5.24)	2498 (12.65)	3431 (16.18)
Always	15 873 (6.66)	10 000 (5.06)	2494 (12.63)	3369 (15.89)
Mean (SD) MET-min per week	2623.13 (2549.46)	2442.18 (2408.33)	3389.44 (2949.94)	3736.84 (3065.37)
Mean (SD) television viewing time	2.40 (1.32)	2.37 (1.29)	2.56 (1.41)	2.63 (1.47)
Mean (SD) portions of fruit/vegetables intake	2.06 (1.43)	2.03 (1.38)	2.09 (1.54)	2.25 (1.70)
Mean (SD) portions of red-meat intake	28 514 (12.01)	22 749 (11.56)	2845 (14.51)	2904 (13.83)
Processed-meat intake >2 times a week [*n* (%)]	75 342 (31.62)	60 666 (30.73)	6551 (33.26)	8113 (38.37)
No oily-fish intake [*n* (%)]	4.03 (2.39)	4.04 (2.32)	4.03 (2.60)	3.96 (2.80)
Mean (SD) units of alcohol intake	17.38 (18.85)	17.20 (18.27)	17.30 (20.40)	19.21 (22.34)
Smoking [*n* (%)]				
Never	139 704 (58.69)	117 216 (59.46)	10 910 (55.38)	11 531 (54.51)
Previous	73 664 (30.95)	61 226 (31.06)	6162 (31.28)	6263 (29.61)
Current	24 672 (10.36)	18 680 (9.48)	2630 (13.35)	3360 (15.88)
Sleep duration <6 hours [n (%)]	10 982 (4.62)	7934 (4.02)	1190 (6.05)	1855 (8.81)
Number of sleep disturbances [*n* (%)]				
0	30 897 (13.12)	26 075 (13.35)	2365 (12.16)	2451 (11.85)
1	101 018 (42.89)	84 973 (43.51)	8011 (41.17)	8018 (38.75)
2	76 186 (32.35)	62 696 (32.10)	6448 (33.14)	7023 (33.94)
3	24 133 (10.25)	19 076 (9.77)	2308 (11.86)	2739 (13.24)
4	3279 (1.39)	2494 (1.28)	324 (1.67)	460 (2.22)
Mean (SD) BMI (kg/m^2^)	27.16 (4.63)	26.99 (4.57)	27.68 (4.90)	28.21 (4.80)
Obesity [*n* (%)]	53 496 (22.49)	41 977 (21.31)	5220 (26.52)	6279 (29.73)
Mean (SD) waist circumference (cm)	89.42 (13.19)	88.92 (13.07)	90.63 (13.65)	92.95 (13.26)
Central obesity [*n* (%)]	124 895 (52.48)	101 381 (51.44)	11 178 (56.77)	12 296 (58.18)
Mean (SD) handgrip strength (kg)	32.88 (11.06)	32.65 (10.99)	32.33 (11.15)	35.57 (11.32)
Mean (SD) systolic blood pressure (mmHg)	135.11 (17.77)	135.02 (17.82)	135.30 (17.79)	135.73 (17.26)
Mean (SD) LDL cholesterol (mmol/L)	3.58 (0.82)	3.58 (0.82)	3.57 (0.82)	3.57 (0.83)
Mean (SD) lipoprotein(a) (nmol/L)	48.94 (59.05)	48.70 (59.00)	50.15 (59.65)	49.97 (58.93)
Mean (SD) HbA1c (mmol/mol)	35.18 (6.12)	35.05 (5.93)	35.69 (6.65)	35.93 (7.14)
Mean (SD) cystatin C (mg/L)	0.87 (0.13)	0.87 (0.13)	0.88 (0.13)	0.88 (0.13)
Mean (SD) GGT (U/L)	35.49 (37.71)	35.02 (37.20)	36.23 (38.33)	39.19 (41.35)

Data presented as mean and SD for continuous variables and as frequency and % for categorical variables. SD, standard deviation; MET, metabolic equivalent of tasks; BMI, body mass index; LDL, low-density lipoprotein; HbA1c, glycated haemoglobin; GGT, gamma-glutamyltransferase.

The associations between shift work and CVD are shown in Table 2. Adjusting for socio-economic and work-related factors attenuated the associations, but shift workers remained more likely to have CVD events (HR 1.11, 95% CI 1.06–1.19) and die from CVD (HR 1.25, 95% CI 1.08–1.44). The associations were further attenuated following adjustment for potential mediators, notably adiposity and serum biomarkers. Similar results were found for incident IHD (HR 1.09, 95% CI 1.03–1.15) and heart failure (HR 1.15, 95% CI 1.03–1.28), but not stroke (HR 1.09, 95% CI 0.99–1.20), after adjustment for socio-economic and work-related factors (Supplementary Table S1, available as Supplementary data at *IJE* online).

**Table 2:** Associations between shift work and cardiovascular disease (CVD) by adjustment models

	Incident CVD		Fatal CVD	
	HR (95% CI)	*P*	HR (95% CI)	*P*
Model 0	1.25 (1.20, 1.30)	<0.0001	1.43 (1.25, 1.63)	<0.0001
Model 1	1.14 (1.09, 1.19)	<0.0001	1.27 (1.11, 1.46)	0.0005
Model 2	1.11 (1.06, 1.16)	<0.0001	1.25 (1.08, 1.44)	0.002
+ PA factors	1.09 (1.04, 1.15)	0.0006	1.24 (1.04, 1.47)	0.02
+ Dietary factors	1.10 (1.05, 1.15)	<0.0001	1.24 (1.07, 1.43)	0.004
+ Smoking/drinking	1.10 (1.05, 1.15)	<0.0001	1.23 (1.06, 1.44)	0.007
+ Sleep factors	1.09 (1.05, 1.14)	<0.0001	1.26 (1.09, 1.46)	0.002
+ Social factors	1.10 (1.06, 1.15)	<0.0001	1.26 (1.09, 1.46)	0.002
+ Adiposity	1.08 (1.03, 1.13)	0.0007	1.22 (1.05, 1.41)	0.008
+ Physical markers	1.10 (1.05, 1.16)	<0.0001	1.21 (1.04, 1.41)	0.01
+ Serum markers	1.08 (1.04, 1.14)	0.0006	1.20 (1.03, 1.40)	0.02

Model 0: age and sex only; Model 1: education and deprivation additionally; Model 2: hours of work per week, duration of current job, walking/standing at work, heavy manual/physical work additionally.

The association between shift work and CVD mortality increased slightly with the frequency of shifts but was not significantly different between evening/weekend and night shifts (Table 3). In the dose-relationship analyses, the association of years of shift work with incident and fatal CVD events increased monotonically (Supplementary Figure S3, available as Supplementary data at *IJE* online). There were significant interactions with sex (*P*_interaction_ = 0.0007) and heavy manual labour (*P*_interaction_ = 0.004) (Table 4). Shift work was more strongly associated with incident CVD in women (HR 1.16, 95% CI 1.07–1.27) and work with minimal heavy manual labour (HR 1.18, 95% CI 1.10–1.27) participants compared with men and work with more heavy manual labour. The association with fatal CVD was also stronger in participants aged >50 years (HR 1.41, 95% CI 1.20–1.65).

**Table 3:** Associations of frequency and type of shift work with cardiovascular disease (CVD)

	Incident CVD		Fatal CVD	
	HR (95% CI)	*P*	HR (95% CI)	*P*
Frequency of shift				
Model 0				
Sometimes	1.23 (1.17, 1.30)	<0.0001	1.37 (1.16, 1.62)	0.0003
Always	1.27 (1.20, 1.35)	<0.0001	1.52 (1.26, 1.83)	<0.0001
Model 1				
Sometimes	1.14 (1.09, 1.20)	<0.0001	1.24 (1.05, 1.47)	0.01
Always	1.13 (1.07, 1.20)	<0.0001	1.32 (1.09, 1.59)	0.004
Model 2				
Sometimes	1.11 (1.05, 1.17)	0.0002	1.21 (1.01, 1.45)	0.04
Always	1.10 (1.03, 1.17)	0.003	1.30 (1.07, 1.58)	0.009
Type of shift				
Model 0				
Evening/weekend shifts	1.25 (1.19, 1.32)	<0.0001	1.44 (1.20, 1.71)	<0.0001
Night shifts	1.25 (1.18, 1.32)	<0.0001	1.43 (1.20, 1.70)	<0.0001
Model 1				
Evening/weekend shifts	1.16 (1.09, 1.22)	<0.0001	1.30 (1.08, 1.55)	0.005
Night shifts	1.12 (1.06, 1.19)	<0.0001	1.25 (1.05, 1.50)	0.01
Model 2				
Evening/weekend shifts	1.13 (1.06, 1.20)	<0.0001	1.29 (1.07, 1.56)	0.01
Night shifts	1.08 (1.02, 1.15)	0.007	1.21 (1.00, 1.46)	0.05

Model 0: age and sex only; Model 1: education and deprivation additionally; Model 2: hours of work per week, duration of current job, walking/standing at work, heavy manual/physical work additionally.

**Table 4:** Association between shift work and cardiovascular disease (CVD) by subgroup

	Incident CVD			Fatal CVD		
	HR (95% CI)	*P*	*P*_interaction_	HR (95% CI)	*P*	*P*_interaction_
Age			0.32			0.02
≤50 years	1.08 (0.98, 1.18)	0.30		0.76 (0.54, 1.08)	0.84	
>50 years	1.11 (1.06, 1.17)	0.002		1.41 (1.20, 1.65)	0.002	
Sex			0.0007			0.82
Female	1.16 (1.07, 1.27)	0.005		1.26 (0.92, 1.71)	0.84	
Male	1.08 (1.02, 1.14)	0.045		1.24 (1.05, 1.46)	0.13	
Education attainment			0.11			0.34
Non-university	1.09 (1.04, 1.14)	0.0084		1.31 (1.12, 1.54)	0.01	
University	1.20 (1.08, 1.32)	0.045		1.01 (0.71, 1.44)	0.96	
Area-based deprivation			0.16			0.98
More deprived	1.13 (1.07, 1.20)	0.002		1.24 (1.03, 1.48)	0.22	
Less deprived	1.07 (1.00, 1.15)	0.26		1.27 (1.00, 1.62)	0.39	
Hours of work per week			0.47			0.23
≤37	1.12 (1.05, 1.19)	0.01		1.14 (0.92, 1.42)	0.96	
>37	1.10 (1.03, 1.17)	0.02		1.32 (1.08, 1.60)	0.07	
Heavy manual labour			0.004			0.72
Never or rarely	1.18 (1.10, 1.27)	0.002		1.16 (0.90, 1.49)	0.96	
Sometimes or more	1.05 (0.99, 1.11)	0.28		1.29 (1.08, 1.54)	0.13	
Chronotype			0.40			0.56
Definitely morning	1.09 (1.00, 1.19)	0.24		1.38 (1.04, 1.82)	0.22	
More morning	1.07 (0.98, 1.17)	0.30		1.12 (0.83, 1.51)	0.96	
More evening	1.10 (1.01, 1.20)	0.21		1.36 (1.04, 1.77)	0.22	
Definitely evening	1.13 (0.97, 1.31)	0.30		1.43 (0.90, 2.28)	0.84	

Adjusted for age, sex, ethnicity, education, deprivation, hours of work per week, duration of current job, walking/standing at work and heavy manual/physical work.

*P*-values for subgroup analyses were corrected using Holm’s Bonferroni procedure.

**Figure 1:**
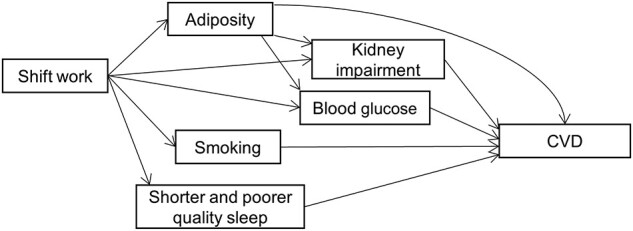
Schematic directed acyclic graph between shift work and cardiovascular disease (CVD) Results based on Table 5. Some correlations (e.g. adiposity and smoking) and confounders were omitted for clarity. The path from sleep disturbances to CVD for incident CVD only. Adiposity could be a causal factor for shift work^26^ despite the current assumption.

Mediation analyses are summarized in Table 5. Current smoking, sleep disturbance, obesity, central obesity, HbA1c and cystatin C were chosen for the mediation analysis of incident CVD because of their associations with both shift work and outcomes. Collectively they explained 52.3% of the association between shift work and incident CVD assuming all correlations between mediators were captured in mutual adjustment. Current smoking (14.1%), sleep (12.3%: 6.2% from short sleep duration and 6.1% from sleep disturbance), adiposity (9.8%: 4.9% from obesity and central obesity, respectively), HbA1c (10.7%) and cystatin C (5.5%) were identified as the main mediators. The results for CVD mortality were generally similar except for sleep factors because they were not associated with CVD mortality. The mediators collectively explained 14.1% of the association between shift work and CVD mortality. Details of the association estimates are shown in Supplementary Tables S2 and S3 (available as Supplementary data at *IJE* online). In the sensitivity analysis, removal of the serum biomarkers increased the proportional contribution of adiposity markers to the mediation (Supplementary Table S4, available as Supplementary data at *IJE* online). A schematic directed acyclic graph based on the mediation analysis results is shown in Figure 1.

**Table 5:** Summary of mediation analyses

	Association with shift work^a^	Incident CVD		Fatal CVD	
		Association with outcome^b^	% mediated (95% CI)	Association with outcome^b^	% mediated (95% CI)
Total^c^			52.3		14.1
MET-min per week	+	0		0	
Hours of TV viewing	0	+		+	
Portions of red-meat intake	+	0		0	
No oily-fish intake	0	+		0	
Frequent processed-meat intake	0	0		0	
Portions of fruit/vegetable intake	+	0		0	
Units of alcohol intake	0	0		0	
Current smoker	+	+	14.1 (7.2–65)	+	8.3 (3.2–43)
Short sleeper	+	+	6.2 (2.4–41)	0	
Sleep disturbance	+	+	6.1 (2.2–35)	0	
Frequency of social visits	0	0		−	
Obesity	+	+	4.9 (1.7–32)	+	2.3 (−8.5, 16)
Central obesity	+	+	4.9 (1.4–23)	+	2.9 (−10.5, 25)
Grip strength	−	−	0.0 (−4.3, 3.0)	−	0.2 (−0.8, 2)
Systolic blood pressure	−	+		+	
LDL cholesterol	0	+		0	
Lipoprotein(a)	0	+		+	
HbA1c	+	+	10.7 (5.0–41)	+	5.8 (2.4–33)
Cystatin C	+	+	5.5 (1.5–28)	+	3.1 (−12, 19)
GGT	0	+		+	

+ positive association; − negative association; 0 no significant association; CVD, cardiovascular disease.

a Summarized from Supplementary Table S2 (available as Supplementary data at *IJE* online); multiple linear or logistic-regression models with the potential mediator as the dependent variable and shift work as the independent variable. Adjusted for each other and for age, sex, education, deprivation, hours of work per week, duration of current job, walking/standing at work and heavy manual/physical work.

b Summarized from Supplementary Table S3 (available as Supplementary data at *IJE* online); Cox regression models with potential mediators as independent variables. Adjusted for each other and for shift work, age, sex, education, deprivation, hours of work per week, duration of current job, walking/standing at work and heavy manual/physical work.

c Total %s mediated were the sum of %s from all significant mediators. This is based on all mediators being mutually adjusted in analyses.

## Discussions

### Principal findings

This study showed that shift workers were at higher risk of incident and fatal CVD events even after accounting for socio-demographic and work-related factors. There was evidence that the associations were cumulative but no strong evidence that the frequency and type of shift modified CVD risk. Over half of the association between shift work and incident CVD could be explained by lifestyle and cardiometabolic factors. These factors, such as adiposity and metabolic status, are largely modifiable and can be addressed by workplace interventions, which have been shown to effective for weight-management and physical-activity outcomes.^27^

To our knowledge, this is the first study to report the association between shift work and heart failure^28^—a CVD with increasing burden globally.^29^ Interestingly, the association of shift work with heart failure was stronger than that with stroke and IHD. This could be due to differential aetiology between these CVDs, which warrants further studies.

### Comparison with other studies

The main findings of this study are generally consistent with the existing evidence. A meta-analysis of prospective studies, conducted in 2018, reported associations between shift work and IHD and CVD mortality with pooled risk ratios (RRs) of 1.26 and 1.17, respectively, but with moderate to high heterogeneity.^7^ Another meta-analysis, also in 2018, identified linear associations between shift work and non-fatal and fatal CVD with pooled RRs of 1.06 and 1.04, respectively, for each 5-year increase in working shifts.^30^ A meta-analysis focusing on IHD reported a pooled RR of 1.13 for shift workers compared with non-shift workers.^31^ However, even though the overall findings were consistent, the estimated effect sizes were not. For example, our study has identified a stronger association for mortality than for incident events, suggesting that shift work impacts prognosis, and provided some evidence of a non-linear relationship between shift work and incident CVD. These differences could be because previous meta-analyses synthesized studies that used different designs and between-study confounders could not be fully adjusted. In addition, the present study also adds to the literature by investigating the type and frequency of shifts, which was not feasible in previous meta-analyses.

The present study is, to our knowledge, the largest study on the mediation between shift work and CVD. It adds empirical evidence to the proposed mechanistic framework linking shift work and CVD.^9^ The three proposed pathways (behavioural, psychosocial and physiological) were tested explicitly in this study but only behavioural (smoking) and physiological (adiposity and cardiometabolic markers) pathways were found to mediate the association between shift work and CVD. A causal pathway between shift work and CVD that operates via obesity and metabolic dysfunction was elucidated from the sensitivity mediation analysis in this study and is supported by previous studies.^32^^,^^33^ These factors could be related to shift workers having higher calorie intake.^34^ We should, however, note that cystatin C, itself, is unlikely to be a causal factor for CVD^17^^,^^18^ but is rather operating as a proxy measure of general kidney dysfunction. An association between shift work and chronic kidney disease has been reported previously.^35^^,^^36^

It was surprising that social activity was not a mediator, given its close relationship with depression and thus CVD.^37^ However, this could be because the frequency of social visits is an imprecise indicator of psychosocial wellbeing and less sensitive to the influence of shift work. We also could not find evidence to support a mediating role for sleep duration (for fatal CVD), physical activity and diet quality (for incident and fatal CVDs). This could be because the effect of these behavioural factors was fully mediated through other mediators (e.g. shift work could cause shorter sleep, which causes adiposity). It is, nonetheless, also possible that the apparent association between sleep and CVD, reported previously,^38^ represented residual confounding from socio-demographic factors for which we were able to control. Shift workers were found to have lower blood pressure than non-shift workers in this study. This could be due to the adjustment of other potential mediators (as in Table 1) or that shift workers might be more likely to be diagnosed and treated for hypertension.

### Strengths and limitations

This study has several strengths over previous studies. First, this is the largest prospective cohort study on shift work and CVD.^7^^,^^30^ Even though it is smaller than a meta-analysis, it has the advantage of consistent study design and measurements.^29^ Conducting analyses of individual-level data ensured consistent methodology and avoided study-level confounders that are common in meta-regression analysis.^39^ Second, the study was based on a general-population cohort covering diverse occupational sectors rather than a specific occupational cohort as used in some previous studies.^40^ This minimized the potential selection bias introduced when studying a single occupation.^41^ To minimize bias resulting from healthier people being preferentially selected to undertake shift work,^11^ we only included participants who did not have major chronic illnesses at baseline. Third, we were able to examine differences between types and frequency of shifts, as well as years working on shift, thereby addressing limitations of many previous studies.^11^ Fourth, we explicitly stated the assumed roles (confounders vs mediators) for each of the covariates and were able to conduct mediation analysis under a counternatural framework to compare the relative importance of the mediators. However, as with any observational study, residual confounding is possible. In the mediation analysis, we adopted a conservative approach in which all mediators and confounders were mutually adjusted. This was likely to underestimate the role of the mediators because of sequential mediation. Owing to this, as well as measurement errors of mediators (lifestyle factors were self-reported), factors that were not proven to be mediators in this study may nonetheless be. Similarly, the contributions of factors that are more ‘upstream’ in the causal chain (e.g. sleep) may be underestimated. Furthermore, mediation analysis assumes causality between shift work and the mediators even though these factors were assessed at the same time in this study. There is evidence showing BMI (but not smoking or alcohol intake) to be causal in participation in shift work.^26^ Therefore, the mediation of adiposity in this study could be overestimated. It should also be noted that shift work and the duration of it were measured in the baseline assessment and could have changed. Nonetheless, this misclassification is unlikely to systemically bias the associations estimated. There were insufficient participants who worked night shifts exclusively to provide a reliable estimate, nor information on the exact hours working in the evening or at night. Future occupational cohorts should explore whether these shifts were associated with CVD differentially. Lastly, whilst the UK Biobank cohort is not representative of the general population in terms of lifestyle.^42^^,^^43^ Therefore, whilst effect sizes should be generalizable, as shown in previous analysis,^44^ summary statistics and estimates of absolute risk should not be generalized.

### Implications of this study

This study addressed limitations in previous studies and provided more robust evidence for the associations between shift work and CVD. The relationships were largely consistent across frequencies and types of shift work, and were stronger among people who had worked shifts for ≥3 years. The mediation analyses suggest that shift work may predispose to smoking and obesity, which, in turn, affect metabolic function and subsequently increase risk of CVD, as outlined in Figure 1. This highlights the need to better manage CVD risk among shift workers, through lifestyle interventions such as smoking and weight management that could be delivered within workplace settings targeted at those most at risk.^27^ Moderation analysis also highlights the groups of people who may be relatively vulnerable to shift work, e.g. those working in non-manual jobs. Future studies could explore the association between combinations of work characteristics and CVD.

## Conclusion

Shift workers have a higher risk of incident and fatal CVD, partly mediated through modifiable risk factors such as smoking, sleep duration and quality, adiposity and metabolic status.

## Supplementary data

Supplementary data are available at *IJE* online.

## Ethics approval

UK Biobank received ethical approval from the North West Multi-Centre Research Ethics Committee (REC reference: 11/NW/03820). All participants gave written informed consent before enrolment in the study, which was conducted in accordance with the principles of the Declaration of Helsinki. Direct dissemination of the results to participants is not possible/applicable.

## Funding

F.K.H. acknowledges University of Glasgow’s Wellcome Trust Institutional Strategic Support Fund Early Career Researcher Catalyst. S.V.K. acknowledges funding from the NHS Research Scotland Senior Clinical Fellowship [grant number SCAF/15/02]; the Medical Research Council [grant number MC_UU_12017/13]; and the Scottish Government Chief Scientist Office [grant number SPHSU13]. The funders played no part in the research. UK Biobank was established by the Wellcome Trust Medical Research Council, Department of Health, Scottish government and the Northwest Regional Development Agency. It has also had funding from the Welsh assembly government and the British Heart Foundation.

## Data availability

Data can be requested from https://www.ukbiobank.ac.uk/.

## Supplementary Material

dyab144_Supplementary_Data
