# Supplementary material for: Association and pathways between shift work and cardiovascular disease: a prospective cohort study of 238 661 participants from UK Biobank
Source: Int J Epidemiol. 2021 Aug 20;51(2):579–90. doi: 10.1093/ije/dyab144 (PMC9082805; doi:10.1093/ije/dyab144)
Supplement: dyab144_Supplementary_Data [file dyab144_supplementary_data.docx]

Association and pathways between shift work and cardiovascular disease: A prospective cohort study of 238,669 participants from UK Biobank

Supplementary materials

**Supplementary Figure S1**. Participant flowchart

All UK Biobank participants

N = 502,493

N = 287,132

Excluded 215,361 participants not in paid employment or self-employed at baseline

N = 286,413

Excluded 719 participants who did not respond to the shift work question

Excluded 47,752 participants who reported chronic conditions at baseline

N = 238,661 included in analysis

**Supplementary Figure S2**. Theoretical direct acyclic graph guiding the analyses


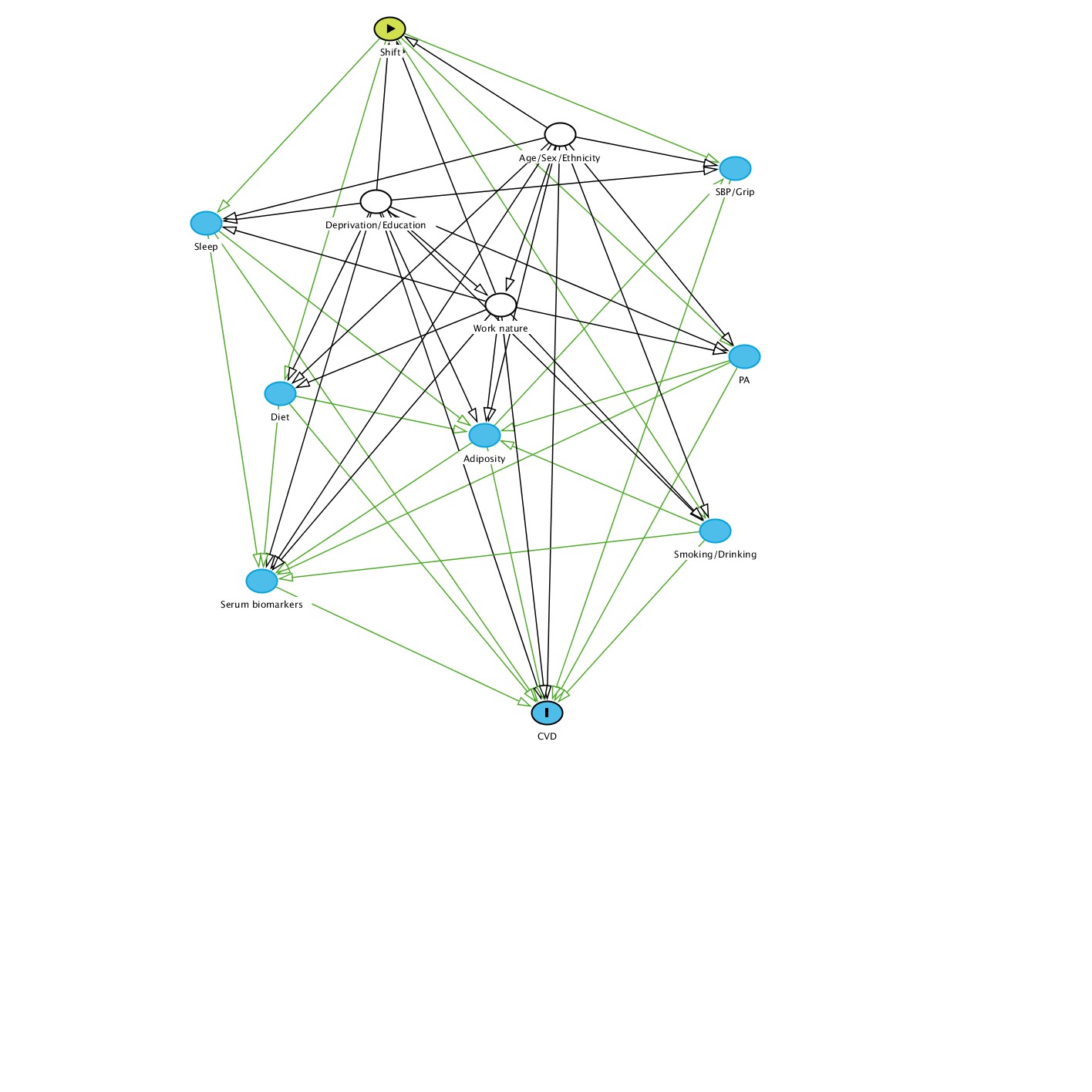


Adiposity could be a causal factor for shift work despite the current assumption.

**Supplementary Figure S3**. Association of years of shift work and incident and fatal CVD


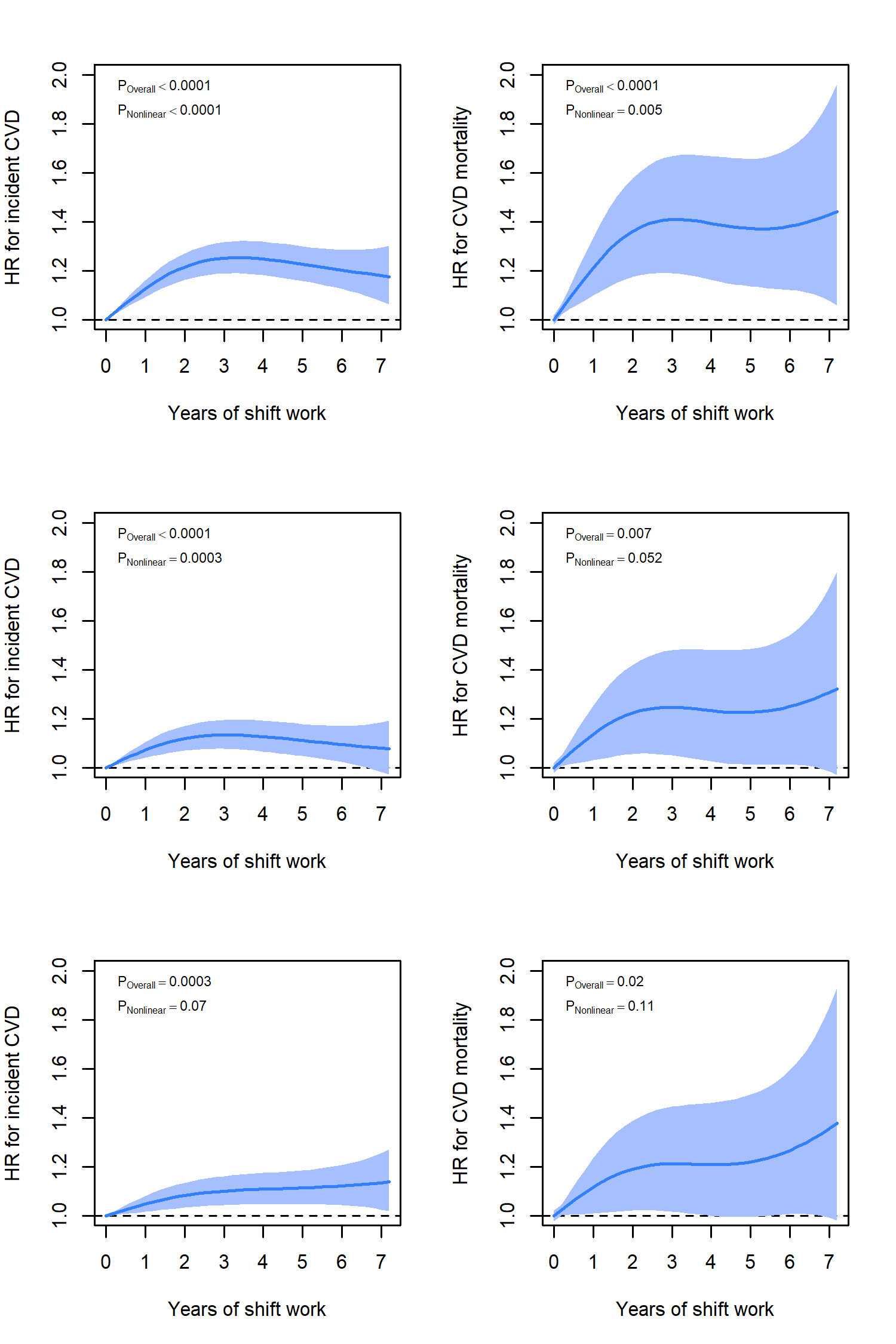


Row 1: Model 0: adjusted for age and sex only;
Row 2: Model 1: adjusted for education and deprivation additionally;
Row 3: Model 2: adjusted for hours of work a week, walking/standing at work, heavy manual/physical work additionally

**Supplementary Table S1**. Association between shift work and types of cardiovascular disease by adjustment models

|  | **IHD** | | **Stroke** | | **Heart failure** | |
| --- | --- | --- | --- | --- | --- | --- |
|  | HR (95% CI) | P | HR (95% CI) | P | HR (95% CI) | P |
| Model 0 | 1.23 (1.17, 1.29) | <0.0001 | 1.18 (1.08, 1.29) | 0.0003 | 1.29 (1.16, 1.42) | <0.0001 |
| Model 1 | 1.11 (1.06, 1.17) | <0.0001 | 1.09 (1.00, 1.20) | 0.054 | 1.15 (1.04, 1.27) | 0.007 |
| Model 2 | 1.09 (1.03, 1.15) | 0.002 | 1.09 (0.99, 1.20) | 0.09 | 1.15 (1.03, 1.28) | 0.01 |
| + PA factors | 1.07 (1.00, 1.13) | 0.047 | 1.11 (1.00, 1.25) | 0.06 | 1.10 (0.97, 1.25) | 0.15 |
| + Dietary factors | 1.08 (1.02, 1.13) | 0.008 | 1.08 (0.98, 1.20) | 0.11 | 1.15 (1.03, 1.28) | 0.01 |
| + Smoking/drinking | 1.08 (1.02, 1.14) | 0.008 | 1.08 (0.97, 1.19) | 0.16 | 1.17 (1.05, 1.31) | 0.006 |
| + Sleep factors | 1.07 (1.02, 1.13) | 0.01 | 1.08 (0.98, 1.19) | 0.13 | 1.13 (1.02, 1.27) | 0.02 |
| + Social factors | 1.08 (1.03, 1.14) | 0.003 | 1.09 (0.99, 1.21) | 0.07 | 1.14 (1.02, 1.27) | 0.02 |
| + Adiposity | 1.06 (1.00, 1.12) | 0.04 | 1.08 (0.98, 1.19) | 0.14 | 1.10 (0.99, 1.23) | 0.08 |
| + Physical markers | 1.08 (1.02, 1.14) | 0.005 | 1.07 (0.96, 1.18) | 0.21 | 1.14 (1.02, 1.27) | 0.02 |
| + Serum markers | 1.08 (1.02, 1.14) | 0.01 | 1.07 (0.97, 1.19) | 0.18 | 1.10 (0.98, 1.23) | 0.1 |

Adjusted for age, sex, ethnicity, education, deprivation, hours of work a week, duration of current job, walking/standing at work, and heavy manual/physical work

IHD: ischaemic heart disease; HR: hazard ratio; CI: confidence interval; PA: physical activity

**Supplementary Table S2**. Associations between shift work and potential mediators

|  | β (95% CI) | P |
| --- | --- | --- |
| **MET-min per week** | **0.03 (0.02, 0.05)** | **< 0.0001** |
| Hours of TV viewing | 0.01 (0.00, 0.03) | 0.06 |
| **Portions of red meat intake** | **0.05 (0.03, 0.07)** | **< 0.0001** |
| No oily fish intake* | -0.03 (-0.08, 0.02) | 0.30 |
| Frequent process meat intake* | 0.04 (0.00, 0.07) | 0.051 |
| **Portions of fruit/vegetable intake** | **0.09 (0.05, 0.12)** | **< 0.0001** |
| Units of alcohol intake | -0.01 (-0.02, 0.00) | 0.15 |
| **Current smoker*** | **0.17 (0.12, 0.22)** | **< 0.0001** |
| **Short sleeper*** | **0.28 (0.22, 0.35)** | **< 0.0001** |
| **Sleep disturbance** | **0.07 (0.05, 0.08)** | **< 0.0001** |
| Frequency of social visits | 0.01 (0.00, 0.03) | 0.13 |
| **Obesity*** | **0.11 (0.06, 0.15)** | **< 0.0001** |
| **Central obesity*** | **0.12 (0.08, 0.16)** | **< 0.0001** |
| **Grip strength** | **-0.03 (-0.04, -0.02)** | **< 0.0001** |
| **Systolic blood pressure** | **-0.03 (-0.04, -0.01)** | **0.0002** |
| LDL cholesterol | -0.01 (-0.03, 0.00) | 0.17 |
| Lipoprotein(a) | 0.01 (-0.01, 0.02) | 0.26 |
| **HbA1c** | **0.04 (0.02, 0.05)** | **< 0.0001** |
| **Cystatin C** | **0.02 (0.00, 0.03)** | **0.01** |
| GGT | 0.00 (-0.02, 0.01) | 0.87 |

β: standardised regression coefficients; MET: metabolic equivalent of tasks; TV: television; LDL: low-density lipoprotein; HbA1c: glycated haemoglobin; GGT: Gamma-glutamyltransferase

* binary variables modelled by logistic regressions; the exponentiation of βs are odds ratios

Adjusted for each other and for age, sex, education, deprivation, hours of work a week, duration of current job, walking/standing at work, and heavy manual/physical work.

**Supplementary Table S3**. Association between all potential mediators and CVD and mediation proportions

|  | Incident CVD | | | | Fatal CVD | | | | |
| --- | --- | --- | --- | --- | --- | --- | --- | --- | --- |
|  | Outcome regressed by potential mediator | | Mediation analysis | | Outcome regressed by potential mediator | | Mediation analysis | |  |
|  | HR (95% CI) | P | % mediated | P_mediation_ | HR (95% CI) | P | % mediated | P_mediation_ |  |
| MET-min per week | 1.01 (0.99, 1.04) | 0.24 | - | - | 1.08 (0.99, 1.18) | 0.07 | - | - |  |
| Hours of TV viewing | 1.04 (1.02, 1.06) | 0.0005 | - | - | 1.08 (1.00, 1.17) | 0.04 | - | - |  |
| Portions of red meat intake | 1.01 (0.99, 1.02) | 0.44 | - | - | 1.00 (0.95, 1.06) | 0.93 | - | - |  |
| No oily fish intake | 1.10 (1.03, 1.18) | 0.004 | - | - | 0.87 (0.67, 1.12) | 0.28 | - | - |  |
| Frequent process meat intake | 1.00 (0.99, 1.01) | 0.47 | - | - | 1.00 (0.96, 1.03) | 0.80 | - | - |  |
| Portions of fruit/vegetable intake | 0.98 (0.94, 1.03) | 0.47 | - | - | 1.06 (0.91, 1.25) | 0.45 | - | - |  |
| Units of alcohol intake | 1.00 (0.98, 1.02) | 0.84 | - | - | 1.06 (1.00, 1.13) | 0.07 | - | - |  |
| **Current smoker** | **1.55 (1.46, 1.65)** | **< 0.0001** | **14.1** | **< 0.0001** | **2.04 (1.68, 2.48)** | **< 0.0001** | **8.3** | **< 0.0001** |  |
| **Short sleeper** | **1.20 (1.09, 1.32)** | **0.0001** | **6.2** | **< 0.0001** | 0.67 (0.45, 1.02) | 0.06 | - | - |  |
| **Sleep disturbance** | **1.06 (1.03, 1.09)** | **< 0.0001** | **6.1** | **< 0.0001** | 1.00 (0.92, 1.08) | 0.94 | - | - |  |
| Frequency of social visits | 1.01 (0.99, 1.03) | 0.41 | - | - | 0.92 (0.86, 0.99) | 0.03 | - | - |  |
| **Obesity** | **1.17 (1.11, 1.24)** | **< 0.0001** | **4.9** | **< 0.0001** | **1.15 (0.96, 1.38)** | **0.14** | **2.3** | **< 0.0001** |  |
| **Central obesity** | **1.12 (1.06, 1.18)** | **< 0.0001** | **4.9** | **< 0.0001** | **1.38 (1.15, 1.66)** | **0.0007** | **2.9** | **< 0.0001** |  |
| Grip strength | 0.90 (0.87, 0.93) | < 0.0001 | 0.0 | 0.94 | 0.85 (0.76, 0.95) | 0.004 | 0.2 | 0.55 |  |
| Systolic blood pressure | 1.20 (1.17, 1.23) | < 0.0001 | - | - | 1.34 (1.25, 1.44) | < 0.0001 | - | - |  |
| LDL cholesterol | 1.07 (1.05, 1.09) | < 0.0001 | - | - | 0.97 (0.90, 1.04) | 0.39 | - | - |  |
| Lipoprotein(a) | 1.12 (1.09, 1.14) | < 0.0001 | - | - | 1.20 (1.12, 1.29) | < 0.0001 | - | - |  |
| **HbA1c** | **1.11 (1.09, 1.12)** | **< 0.0001** | **10.7** | **< 0.0001** | **1.16 (1.13, 1.20)** | **< 0.0001** | **5.8** | **< 0.0001** |  |
| **Cystatin C** | **1.14 (1.12, 1.17)** | **< 0.0001** | **5.5** | **< 0.0001** | **1.24 (1.17, 1.30)** | **< 0.0001** | **3.1** | **< 0.0001** |  |
| GGT | 1.03 (1.02, 1.05) | < 0.0001 | - | - | 1.06 (1.02, 1.11) | 0.006 | - | - |  |

CVD: cardiovascular disease; HR: hazard ratio; CI: confidence interval; MET: metabolic equivalent of tasks; TV: television; LDL: low-density lipoprotein; HbA1c: glycated haemoglobin; GGT: Gamma-glutamyltransferase

* binary variables modelled by logistic regressions; the Adjusted for each other, and for shift work, age, sex, ethnicity, education, deprivation, hours of work a week, duration of current job, walking/standing at work, and heavy manual/physical work

**Supplementary Table S4**. Association between potential mediators (excluding serum biomarkers) and CVD and mediation proportions

|  | Incident CVD | | | | CVD mortality | | | | |
| --- | --- | --- | --- | --- | --- | --- | --- | --- | --- |
|  | Association with outcome | | Mediation analysis | | Association with outcome | | Mediation analysis | | |
|  | HR (95% CI) | P | % mediated | P_mediation_ | HR (95% CI) | P | % mediated | P_mediation_ |  |
| MET-min per week | 1.00 (0.98, 1.03) | 0.7 | - | - | 1.07 (0.98, 1.16) | 0.12 | - | - |  |
| Hours of TV viewing | 1.04 (1.02, 1.07) | 0.0001 | - | - | 1.08 (1.01, 1.16) | 0.03 | - | - |  |
| Portions of red meat intake | 1.01 (0.99, 1.02) | 0.44 | - | - | 1.00 (0.95, 1.05) | 0.88 | - | - |  |
| No oily fish intake | 1.12 (1.05, 1.19) | 0.0007 | - | - | 0.96 (0.76, 1.21) | 0.74 | - | - |  |
| Frequent process meat intake | 1.00 (0.99, 1.00) | 0.26 | - | - | 0.99 (0.96, 1.02) | 0.64 | - | - |  |
| Portions of fruit/vegetable intake | 0.99 (0.95, 1.03) | 0.62 | - | - | 1.12 (0.96, 1.30) | 0.15 | - | - |  |
| Units of alcohol intake | 0.99 (0.97, 1.01) | 0.41 | - | - | 1.05 (0.99, 1.11) | 0.12 | - | - |  |
| **Current smoker** | **1.66 (1.57, 1.76)** | **< 0.0001** | **14.6** | **< 0.0001** | **2.33 (1.94, 2.79)** | **< 0.0001** | **9.7** | **< 0.0001** |  |
| Short sleeper | **1.21 (1.11, 1.32)** | **< 0.0001** | **6.1** | **< 0.0001** | 0.70 (0.48, 1.02) | 0.06 | - | - |  |
| **Sleep disturbance** | **1.07 (1.05, 1.10)** | **< 0.0001** | **6.6** | **< 0.0001** | **1.00 (0.93, 1.08)** | **0.97** | - | - |  |
| Frequency of social visits | 1.01 (0.99, 1.03) | 0.39 | - | - | 0.92 (0.86, 0.98) | 0.01 | - | - |  |
| **Obesity** | **1.26 (1.19, 1.32)** | **< 0.0001** | **7.6** | **< 0.0001** | **1.29 (1.09, 1.53)** | **0.004** | **4.0** | **< 0.0001** |  |
| **Central obesity** | **1.19 (1.13, 1.25)** | **< 0.0001** | **6.8** | **< 0.0001** | **1.52 (1.28, 1.80)** | **< 0.0001** | **4.1** | **< 0.0001** |  |
| Grip strength | 0.90 (0.87, 0.93) | < 0.0001 | 0.0 | 0.33 | 0.81 (0.73, 0.90) | < 0.0001 | 0.4 | 0.55 |  |
| Systolic blood pressure | 1.21 (1.19, 1.24) | < 0.0001 | - | - | 1.34 (1.25, 1.43) | < 0.0001 | - | - |  |

CVD: cardiovascular disease; HR: hazard ratio; CI: confidence interval; MET: metabolic equivalent of tasks; TV: television

Adjusted for each other, and for shift work, age, sex, ethnicity, education, deprivation, hours of work a week, duration of current job, walking/standing at work, and heavy manual/physical work
